# Biochemical effects of irbesartan in experimental diabetic nephropathy

**DOI:** 10.4103/0253-7613.59922

**Published:** 2009-12

**Authors:** Richa Vaishya, J. Singh, Harbans Lal

**Affiliations:** Department of Biochemistry, Pt. BD Sharma Postgraduate Institute of Medical Science, Rohtak, Haryana, India; 1 Department of Pharmacology, Pt. BD Sharma Postgraduate Institute of Medical Science, Rohtak, Haryana, India

**Keywords:** Diabetic nephropathy, irbesartan, streptozotocin

## Abstract

**Background:**

Diabetic nephropathy (DN) is one of the most common causes of end-stage renal failure. The pathogenesis of progressive renal damage is multifactorial and the mechanism by which hyperglycemia causes microangiopathy in diabetic glomeruli is still poorly understood. Because the renin angiotensin system has been reported to be an important contributory factor in the pathophysiology of DN, exogenous administration of angiotensin II receptor antagonist may be beneficial in counteracting some biochemical or functional changes of DN.

**Aims:**

The present study was therefore undertaken to evaluate the preventive role of irbesartan in streptozotocin (STZ)-induced DN in rats.

**Methods and material:**

STZ-induced DN in rats was assessed biochemically by measuring urine volume, protein and electrolytes as well as blood urea and creatinine clearance.

**Results:**

Marked hyperglycemia, polyuria, proteinuria and uremia along with a reduction in urine electrolytes and creatinine clearance were observed in STZ diabetic rats. Pre-treatment with irbesartan (20 mg/kg, p.o. 5 days prior to STZ and continued for 16 weeks) also significantly altered these parameters towards normal, except blood glucose.

**Conclusion:**

Pre-treatment with insulin reversed the parameters of DN. The data suggest that irbesartan prevents the development of STZ-induced DN in rats.

## Introduction

Diabetic nephropathy (DN) is one of the most common causes of end-stage renal failure. It is widely appreciated that normalization of the blood glucose level and vigorous treatment of hypertension retard nephropathic changes.[1] It is also known that many patients with apparently excellent metabolic control have serious complications, whereas those with overtly poor control may be free from clinical problems for many years.[2] Because several factors may contribute to the development of tissue damage, strict control of blood glucose level has not yet been shown to prevent or reverse progressive kidney disease in humans.[3] The pathogenesis of progressive renal damage is multifactorial and the mechanism by which hyperglycemia causes microangiopathy in diabetic glomeruli is still poorly understood. Renal damage may be triggered and sustained by a combination of hemodynamic (increased glomerular pressure and flow), metabolic (hyperglycemia, dyslipidemia and hyperphosphatemia), humoral (angiotensin II and endothelins) and immunological mechanisms.[4] Till date, irrespective of the pathogenesis, there is no satisfactory treatment of progressive DN other than attempting to decrease the symptoms by treatment with angiotensin converting enzyme inhibitors, restriction of dietary protein and treatment of hyperglycemia and dyslipidemia.[5] Recently, irbesartan (an angiotensin II receptor antagonist) has been used as an antihypertensive agent.[6] Because the renin angiotensin system has been reported to be an important contributory factor in the pathophysiology of DN, exogenous administration of angiotensin II receptor antagonist may be beneficial in counteracting some biochemical or functional changes of DN. The present study was therefore undertaken to evaluate the preventive role of irbesartan in streptozotocin (STZ)-induced DN in rats.

## Material and Methods

### Animals used

Adult albino rats, of either sex, weighing between 250 and 300 g were maintained under standard conditions with food and water *ad libitum*. The study was approved by the Institutional Animal Ethical Committee.

### Grouping and treatment

Animals were divided into four groups of 10 each. Group I served as non-diabetic control and received citrate buffer. Animals in group II were made diabetic with STZ (Sigma Chemicals, St Louis, Missouri, USA) with a single injection of 50 mg/kg i.v. (in 0.05 mol/L citrate buffer, pH 4.5). Group III received irbesartan (Sun Pharma, Mumbai, India) 5 days prior to STZ (20 mg/kg, p.o., daily) and continued for 16 weeks along with STZ (as to group II). In group IV, STZ (as to group II) was given along with regular insulin (Sarabhai, Baroda, India) (4 U/kg, s.c., twice daily) for 16 weeks to make these rats euglycemic.

### Biochemical analysis

Samples for glucose monitoring were obtained from the tail vein after 48 h of STZ and every 4 weeks thereafter, up to 16 weeks. Blood glucose was determined by the glucose oxidase method.[7] Rats showing serum glucose levels >250 mg/dl, after 48 h of STZ, were considered diabetic. The extent of DN was assessed biochemically by measuring the urine volume, protein and electrolytes. In addition, blood urea and serum and urine creatinine were estimated and creatinine clearance (GFR) was calculated. Each rat was housed individually in a metabolic cage and urine volume was measured for 6 h Urine proteins were measured using biuret reagent after precipitation.[8] Urinary electrolytes (Na+ and K+) were estimated by flame photometry. Blood urea was estimated by the diacetyl monoxime method.[9] Serum and urine creatinine were determined by modified Jaffe's reaction.[10] Results were statistically analyzed by Student's *t*-test (unpaired). A value of *P* < 0.01 was considered as highly significant.

## Results

The mean blood glucose value in the control group was 90.5 ± 1.17 mg/dl. There was a sustained increase in blood glucose in animals treated with STZ. STZ-induced diabetes in rats produced hyperglycemia within 48 h and the levels remained more than 260 mg/dl throughout the experimental period of up to 16 weeks. Irbesartan pre-treatment failed to influence the blood sugar levels in STZ diabetic rats. However, insulin pre-treatment completely prevented the development of STZ-induced hyperglycemia in rats. Mean urinary output in normal rats was in the range of 3.5 ± 0.19 to 4.3 ± 0.09 ml/6 h. On the other hand, a marked and sustained increase in urine volume was observed in diabetic animals throughout the experimental period. However, polyuria was more marked in the early weeks of diabetes (mean urine output 14.4 ± 0.33 ml/6 hr at 4 weeks) and gradually declined (6.4 ± 0.55 ml/6 h at 16 weeks), although it was significantly higher than the control group. STZ-induced diuresis returned towards normal by pre-treatment with irbesartan and insulin. There was a slight decrease in urinary Na^+^ and K^+^ excretion in STZ-treated diabetic rats. Insulin as well as irbesartan prevented the decrease in electrolyte excretion and the values became comparable to that of controls. There was no proteinuria in the control groups. A sustained increase in urinary protein excretion was observed in diabetic rats (*P* < 0.01). Although insulin pre-treatment completely prevented proteinuria, irbesartan pre-treatment significantly reduced the urinary excretion of protein in these animals [Table 1].

**Table 1:** Effect of irbesartan (20 mg/kg/p.o.) pre-treatment on urinary protein excretion in STZ-induced diabetic rats

*Group*	*Urine protein (g/L)*				
	
	*0 Week*	*4 Week*	*8 Week*	*12 Week*	*16 Week*
I Control	Nil	Nil	Nil	Nil	Nil
II STZ	Nil	1.0 ± 0.07	1.6 ± 0.16	1.9 ± 0.16	2.1 ± 0.13
III Insulin + STZ	Nil	57.3 ± 2.2	70.3 ± 3.3	65.8 ± 2.5	61.8 ± 2.0
IV Irbesartan + STZ	Nil	0.5 ± 0.04‡	0.8 ± 0.06‡	0.9 ± 0.08‡	0.8 ± 0.09‡

Statistically analyzed by Student's t-test (unpaired),

‡ *P* < 0.01 when compared with group II, values are mean ± SEM; n = 10 in each group

Increased blood urea levels were observed in STZ diabetic rats as compared with controls. Insulin as well as irbesartan pre-treatment markedly reduced the elevated levels of blood urea in these rats (*P* < 0.01) [Table 2]. STZ treatment resulted in a gradual decline in GFR (creatinine clearance). Creatinine clearance was improved following pre-treatment with insulin as well as irbesartan [Table 3].

**Table 2:** Effect of irbesartan (20 mg/kg, p.o.) pre-treatment on blood urea levels in STZ-induced diabetic rats

*Group*	*Blood Urea (mg/dl)*				
	
	*0 Week*	*4 Week*	*8 Week*	*12 Week*	*16 Week*
I Control	18.0 ± 0.52	20.8 ± 0.84	19.9 ± 0.60	21.3 ± 0.86	21.6 ± 0.98
II STZ	17.5 ± 0.69	68.7 ± 1.57*	106.0 ± 3.40*	136.0 ± 7.77*	150.0 ± 8.43*
III Insulin + STZ	19.3 ± 0.77	21.2 ± 0.78‡	22.5 ± 0.73‡	23.6 ± 0.50‡	24.4 ± 0.58‡
IV Irbesartan + STZ	17.7 ± 0.63	39.9 ± 0.48‡†	37.9 ± 0.48‡†	35.5 ± 1.02‡†	32.0 ± 0.79‡†

Statistically analyzed by Student's t-test (unpaired),

‡ *P*<0.01 when compared with group II, values are mean ± SEM; n = 10 in each group

**Table 3:** Effect of irbesartan (20 mg/kg, p.o.) pre-treatment on creatinine clearance in STZ-induced diabetic rats

*Group*	*Creatinine clearance (ml/min)*				
	
	*0 Week*	*4 Week*	*8 Week*	*12 Week*	*16 Week*
I Control	1.1 ± 0.18	20.8 ± 0.84	19.9 ± 0.60	21.3 ± 0.86	1.2 ± 0.06
II STZ	0.9 ± 0.07	1.8 ± 0.16	0.9 ± 0.12	0.2 ± 0.11*	0.1 ± 0.03*
III Insulin + STZ	0.9 ± 0.08	0.9 ± 0.07‡	0.8 ± 0.06	0.8 ± 0.08‡	0.7 ± 0.03‡
IV Irbesartan + STZ	1.0 ± 0.03	0.7 ± 0.03‡	37.9 ± 0.48‡†	0.5 ± 0.15	0.4 ± 0.02‡†

Statistically analyzed by Student's t-test (unpaired),

* *P*<0.01 when compared with the control (group I),

‡ *P*<0.01 when compared with group II,

† *P*<0.01 when compared with group III, values are mean ± SEM; n = 10 in each group

## Discussion

Results of the present study confirm that STZ, which is commonly used as a diabetogenic agent in experimental animals,[11] causes hyperglycemia, polyuria, macroproteinuria as well as decrease in GFR. Under such conditions, hyperglycemia is a result of damage to the beta cells. Insulin is frequently used to reverse the changes.[12] One of the long-term complications of diabetes is nephropathy. Microalbuminuria and hypertension are the risk factors for DN. It has been reported that blockage of the renin–angiotensin system slows the progression to DN in patients with type 1 diabetes.[13] Some recent studies have also demonstrated the renoprotective effect of the angiotensin II receptor antagonist, irbesartan, in hypertensive patients.[14] The results of the present study indicate that irbesartan is beneficial in preventing experimental DN in rats, which excreted a large amount of protein in their urine. The beneficial effects of irbesartan in these rats with macroproteinuria may be due to blockade of impact of angiotensin II on blood pressure, renal hemodynamics and non-hemodynamic action of angiotensin II by blockade of growth-promoting, profibrotic and other actions. The mechanism of renoprotection by irbesartan may, however, be complex, involving hemodynamic factors that lower intraglomerular pressure.[15] It is speculated that intrarenal generation of angiotensin II constricts renal efferent arterioles and causes an increase in glomerular hydraulic pressure. Glomerular hyperfilteration and hypertension may then initiate and induce glomerular lesions.[16] Hence, the angiotensin II receptor antagonist irbesartan is expected to cause renal vasodilatation. Chronic treatment with irbesartan in Fawn-hooded hypertensive rats has also been shown to normalize systemic blood pressure and glomerular capillary hydraulic pressure, which prevented the development of proteinuria and glomerulosclerosis.[15]

Recent evidence suggests that nephrin located in the slit diaphragm of the glomerular podocyte could also play a key role in the function of the glomerular filtration barrier and the development of proteinuria.[17] These workers have demonstrated a significant reduction in both gene and protein expression of nephrin in long-term spontaneously hypertensive rats. These changes in nephrin levels were completely prevented by angiotensin receptor blockers. It is possible that some of the renoprotective effect of irbesartan may also be mediated via the AT2 receptor.[18] Besides its renoprotective effect on massive proteinuria, the drug also affects other biochemical and metabolic functions, suggesting that this angiotensin II receptor blocker should be regarded as an important renoprotective agent.

## References

[1] Jenkins DA, Cowan P, Coolier A, Watson ML, Clarke BF (1990). Blood glucose control determines the renal hemodynamic response to angistensin converting enzyme inhibition in type I diabetes. Diabetic Med.

[2] Thomas PK (1986). Diabetic neuropathy, human and experimental. Drugs.

[3] Friedman EA, Friedman EA, Charles M, Paterson (1985). Diabetic Nephropathy: Strategy for therapy.

[4] Varun SN, Arun K (2000). Reno protection with angiotensin type I receptor blockers in hypertension. Indian heart J.

[5] Krolewski AS, Warran JH, Christlieb AR (1994). Hypercholesterolaemia – A determinant of renal function loss and deaths in IDDM and nephropathy. Kidney Int.

[6] Gustav GB, Raunhild B, Susan K, Christian M, Ernst M (1999). Time course and extent of angiotensin II antagonism after irbesatran, losartan and vatsartan in humans assessed by angiotensin II dose response and radioligant and receptor assay. Clin Pharmacol Ther.

[7] Trinder P (1969). Determination of glucose in blood using glucose oxidase with an alternative oxygen acceptor. Ann Clin Biochem.

[8] Johnson MA, Rohlfs EM, Silverman LM, Burtis CA, Ashwood ER (1999). Determination of protein in urine. Teitz Textbook of Clinical Chemistry.

[9] Marsh WH, Fingerhut B, Miller H (1965). Automated and manual direct methods for the determination of blood urea. Clin Chem.

[10] Newman DJ, Price CP, Burtis CA, Ashwood ER (1999). Renal function and nitrogen metabolites. Teitz Textbook of Clinical Chemistry.

[11] Tanya MO, Yunzia YU, Siama P, Steven P, Zafira K, Rober NP (2000). Prevention of albuminuria by aminoguanidine or ramipril in Streptozotocin –induced diabetic rats is associated with the normalization of glomerular protein kinase C. Diabetes.

[12] Papaccio G, Chieffi BG, Mezzogiorno V, Esposito V (1993). Extra islet infiltration in NOD mouse: observations after immunomodulation. Pancreas.

[13] Parving HH, Lehnert H, Brochnr-Mortensh J, Gomis R, Anderson S, Arner P (2001). The effect of irbesartan on the development of diabetic nephropathy in patients with type2 diabetes. N Engl J Med.

[14] Sasso FC, Carbonara O, Persico M, Iafusco D, Salvatore T, D' Ambrossio R (2002). Irbesartan reduces the albumin excretion rate in microalbuminuric type 2 diabetic patients independently of hypertension: a randomized double-blind placebo controlled crossover study. Diabetes Care.

[15] Ziai F, Ots M, Provoost AP, Troy JL, Reenke HG, Brenner BM (1996). The angiotensin receptor antagonist, irbesartan, reduces renal injury in experimental chronic renal failure. Kidney Int.

[16] Cooper ME (2001). Interaction of metabolic and hemodynamic factors in mediating experimental diabetic nephropathy. Diabetologia.

[17] Davis BJ, Cao Z, Gasparo MD, Kawachi H, Cooper ME, Allen TJ (2003). Disparate effects of angiotensin II antagonist and calcium channel blockers on albuminuria in experimental diabetes and hypertension: Potential role of nephrin. J Hypertens.

[18] Tsutsumi Y, Matsubara H, Masaki H, Kurihara H, Murasawa S, Takai S (1999). Angiotensin II type 2 receptor over expression activates the vascular kinin system and causes vasodilatation. J Clin Invest.

